# Update on mosquito bite reaction: Itch and hypersensitivity, pathophysiology, prevention, and treatment

**DOI:** 10.3389/fimmu.2022.1024559

**Published:** 2022-09-21

**Authors:** Ashley Vander Does, Angelina Labib, Gil Yosipovitch

**Affiliations:** Miami Itch Center, Dr Phillip Frost Department of Dermatology, University of Miami, Miami, FL, United States

**Keywords:** hypersensitivity, insect bite, repellant, itch, mosquito, mosquito allergy, genetic predisposition

## Abstract

Mosquito bites are endured by most populations worldwide. Reactions to mosquito bites range from localized wheals and papules with associated pruritus to rare systemic reactions and anaphylaxis in certain populations. The mechanism of itch is due to introduction of mosquito saliva components into the cutaneous tissue, although the exact pathophysiology is unclear. Histamine is thought to be a key player through mosquito saliva itself or through activation of mast cells by IgE or through an IgE-independent pathway. However, other salivary proteins such as tryptase and leukotrienes may induce non-histaminergic itch. Some individuals have a genetic predisposition for mosquito bites, and people with hematologic cancers, HIV, and other conditions are susceptible to robust reactions. Prevention of mosquito bites is key with physical barriers or chemical repellents. Treatment consists of second-generation antihistamines and topical corticosteroids. Further research on topical treatments that target neural-mediated itch is needed.

## Introduction

Mosquitos are ubiquitous and are responsible for most insect bites worldwide (1). Their bite causes a local cutaneous reaction leading to acute pruritus and the subsequent consequences of scratching: scarring, hyperpigmentation, and superinfection. In some individuals, this local cutaneous reaction is exaggerated and debilitating, worsening the clinical course and decreasing quality of life, especially if mosquito bites are a common occurrence.

## Epidemiology

Mosquitos are found in all continents except Antarctica (1). While the incidence of mosquito bites is unknown due to lack of reporting, the largest populations of mosquitos reside in humid tropical regions such as Thailand, Brazil, Indonesia, and the Philippines. Due to global warming, the incidence of mosquito bites is expected to increase as more extensive growth occurs.

There are over 3,500 species and subspecies of mosquito in 42 genera, three genera of which cause human bites: *Anopheles*, *Culex*, and *Aedes* (1, 2). Feeding behavior varies with genus: for instance, *Culex* mosquitos are active mostly at night, whereas the *Aedes* genus is active during the day. Only female mosquitos bite humans, as blood provides the nutrients required to produce eggs (1). Mosquitos find their human or animal host *via* visual color cues, such as dark-colored objects (1, 3). As they draw nearer to the host, they increasingly rely on thermal and olfactory stimuli. Research has shown that mosquitos are particularly drawn to moist heat sources, exhaled carbon dioxide, and certain body odors (4).

The diseases transmitted by mosquitos include malaria (*Anopheles*); West Nile virus and western/eastern equine encephalitis (*Culex*); and Chikungunya, yellow fever, dengue, and the Zika virus (*Aedes*). The burden of mosquito-borne diseases is significant: 700 million infections leading to one million deaths every year (5).

## Clinical features

Mosquito bite responses occur in phases: the immediate reaction and delayed reaction, along with large local reactions in some individuals. Immediately after a mosquito bite, a round wheal 2-10 mm in diameter forms with surrounding erythema peaking in 20-30 minutes (6–8). The delayed reaction consists of pruritic papules of the same size which peak in 24-36 hours. These gradually disappear over the course of several days.

Mosquito bites in human skin progress through a series of stages determined by cumulative number of mosquito bites accrued during a lifetime (6, 9). The first mosquito bite in an individual results in a small, red spot (stage I). Subsequent bites lead to first a delayed reaction only (stage II), then an immediate and delayed reaction (stage III), then an immediate reaction only (stage IV), and finally neither an immediate nor delayed reaction (stage V). Thus, it’s understood that natural desensitization to mosquito saliva may occur with long-term exposure (10), though a subsequent observational study noted marked individual variability in course of the stage progression with 6 of 10 patients remaining in stage III over a 30-year period (11).

For some individuals, a large local reaction (wheal > 5 mm) occurs within minutes to hours (12). These individuals may be diagnosed with a mosquito allergy. In general, mosquito bite size is correlated to self-reported itch intensity (13). Secondary skin lesions due to scratching include excoriations which may obstruct primary skin findings, along with scarring and hyperpigmentation.

Finally, the diseases transmitted by mosquitos and their treatments may also induce pruritus. For instance, the Zika, West Nile, Chikungunya, and dengue viruses cause a generalized maculopapular rash that is often itchy (14–17). Additionally, the anti-malarial drug chloroquine is well-known for causing pruritus (18).

## Predisposition to mosquito bites: Genetics and the skin microbiome

Numerous studies suggest a human susceptibility to mosquito bites and associated itch (19). Khan et al. reported that individuals attracted mosquitos with varying rates and demonstrated differing mosquito bite responses (20). Twin studies of monozygotic and dizygotic twins suggested a strong genetic association to bite susceptibility, possibly due to body odors derived from shared genetics between identical twins that are subsequently detected by mosquito olfaction (19, 21–23). Additionally, self-reported size of the mosquito bite, intensity of the related itch, and perceived attractiveness to mosquitos seemed to have a hereditary component and were greater in females compared to males (13). The sex difference is thought to be due to a specific locus of genes in the human leukocyte antigen (HLA) region that yields a more intense itch response in females to a 3-fold effect (13, 24).

Human body odor results in part from volatile organic compounds emitted by the skin’s commensal bacteria (25). Many studies have shown a relationship between skin bacteria composition and mosquito attractiveness, including direct attractiveness of mosquitos to odors produced by these bacteria (24, 26–28). For example, low diversity of the skin microbiome is correlated with higher attraction rates and *Pseudomonas* spp. is associated with decreased attraction rates (24, 28). Future clinical opportunities may involve manipulating the composition of skin bacteria through application of a topical probiotic, a technique already emerging for management in other disease entities (i.e., psoriasis, eczema). Before microbiome alteration becomes an option, extensive research must answer remaining questions regarding its feasibility, including the duration of treatment effect (25).

## Mosquito itch pathophysiology

**Figure 1:**
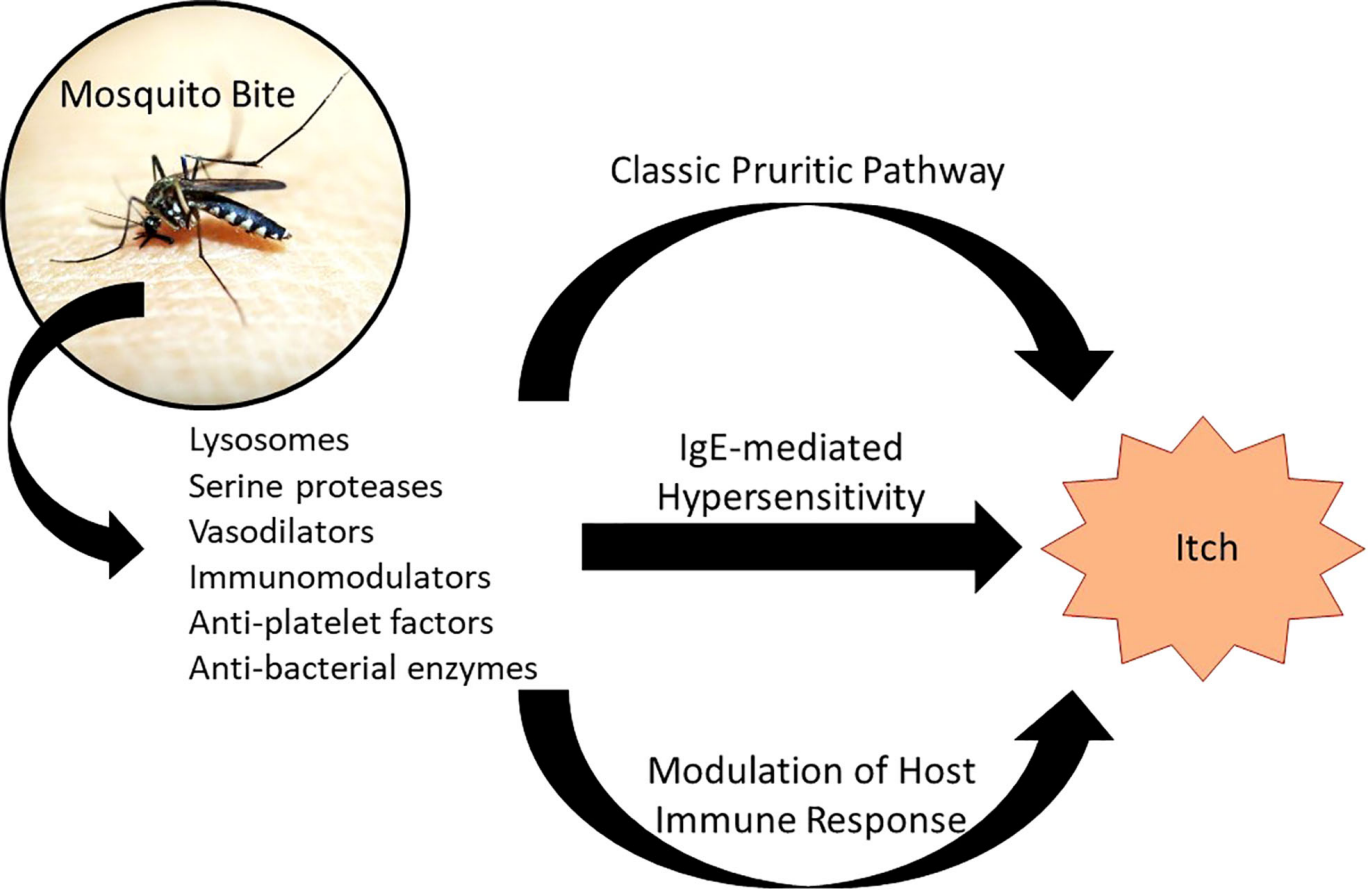
Pathophysiology of Mosquito Bite Itch. Introduction of mosquito saliva components results in a number of local responses, three of which are proposed to cause itch: (1) the classic pruritic pathway involving histamine found in mosquito saliva binding to histamine receptors on sensory nerve endings; (2) an IgE-mediated hypersensitivity, in which IgE primed against mosquito saliva components crosslinks with mast cells, causing degranulation; and (3) modulation of the host immune response through IgE-independent and non-histaminergic pathways. Adapted from: Fostini et al. (29).

While the mechanism of mosquito bite reaction isn’t well-understood, a few hypotheses elucidate the responses occurring from cutaneous introduction of mosquito saliva components: a reaction to histamine found directly in mosquito saliva, an IgE-mediated (type I) hypersensitivity reaction, and an IgE-independent inflammatory response (**Figure 1**) (29).

A histaminergic response is a well-supported mechanism of reaction. Similar to other pruritic pathways like urticaria and mastocytosis, histamine found in mosquito saliva causes itch by binding to histamine-specific receptors on nerve endings (30). Histamine also instigates local vasodilation and edema, causing wheal formation. The amount of histamine found in mosquito saliva appears to be sufficient to induce an itch response. This mechanism is supported by reduction of wheal size and pruritus when patients are treated with anti-histaminergic medications.

Additionally, endogenous histamine is released through IgE activation of mast cells within the dermis in response to other mosquito saliva components (type 1 hypersensitivity). These mosquito saliva components include salivary odorant-binding proteins (Aed a 2, Aed al 2, Ano d 2, Cul q, Cul q 3) and other various proteins (Aed a 1, Aed a 3, Aed a 4, Aed al 3) (31). The D7 proteins, which are a subtype of odorant-binding proteins and abundant in mosquito saliva, were found to be the major allergenic proteins across mosquito species (31). They have been shown to bind to biogenic amine and leukotriene, effectively neutralizing their activity to inhibit host immune defenses and temporarily prevent scratching, which would interrupt feeding (32). Activation of mast cells by IgE primed against these allergens leads to the release of various inflammatory mediators, such as histamine (discussed earlier), cytokines, tryptase, and eicosanoids (such as leukotrienes). Kuraishi et al. studied the effects of various medications on scratching behavior in mice injected with mosquito salivary gland extract (33). They found that drugs which inhibit 5-lipoxygenase (like zileuton) inhibited theincreased activity of the cutaneous nerve branch induced by the extract and decreased scratching, while drugs modifying leukotriene B(4) and cysteinyl leukotrienes (LTC4, LTD4, and LTE4) had no such effect. Therefore, they deduced that 5-lipoxygenase metabolite(s) other than leukotriene B(4) and cysteinyl leukotrienes are involved in mosquito-associated itching. In addition to IgE, mosquito-saliva-specific IgG levels are also elevated during immediate and delayed reactions.

Another proposed mechanism for mosquito itch includes the IgE-independent inflammatory response, implicated in delayed reactions. This occurs through either direct stimulation of mast cells by saliva components resulting in degranulation and/or through a Th2 inflammatory cascade. Indeed, a murine model study by Demeure et al. showed that mosquito saliva can activate mast cells independent of IgE or IgG antibodies directed against salivary components (34). Future research should elicit which mosquito saliva component(s) or mast cell receptor(s) are responsible (34).

Studies have shown that SAAG-4 and sialokinin, proteins/peptides found in mosquito saliva, can induce interleukin (IL)-4 expression and decrease IFN-γ expression, driving the host immune response from a Th1 to a Th2-mediated response (35–37). The release of IL-4 and other cytokines of Th2-mediated responses such as IL-5, IL-13, and IL-31 are known players in itch responses, associated with other pruritic conditions like atopic dermatitis and urticaria (38–41).

## Amplified mosquito bite reactions

Certain populations exhibit an increased reaction to mosquito bites beyond typical mast cell degranulation including children, outdoor workers with a high degree of exposure, and those with no previous exposure to indigenous mosquitoes (42). These rare, exaggerated responses also occur in several conditions, especially immune disorders. In general, a diagnosis of mosquito hypersensitivity is based on patient history; commercially available skin prick testing for whole body mosquito allergen extract is available only in select countries and does not have a significant role in patient evaluation.

### Children

**Figure 2:**
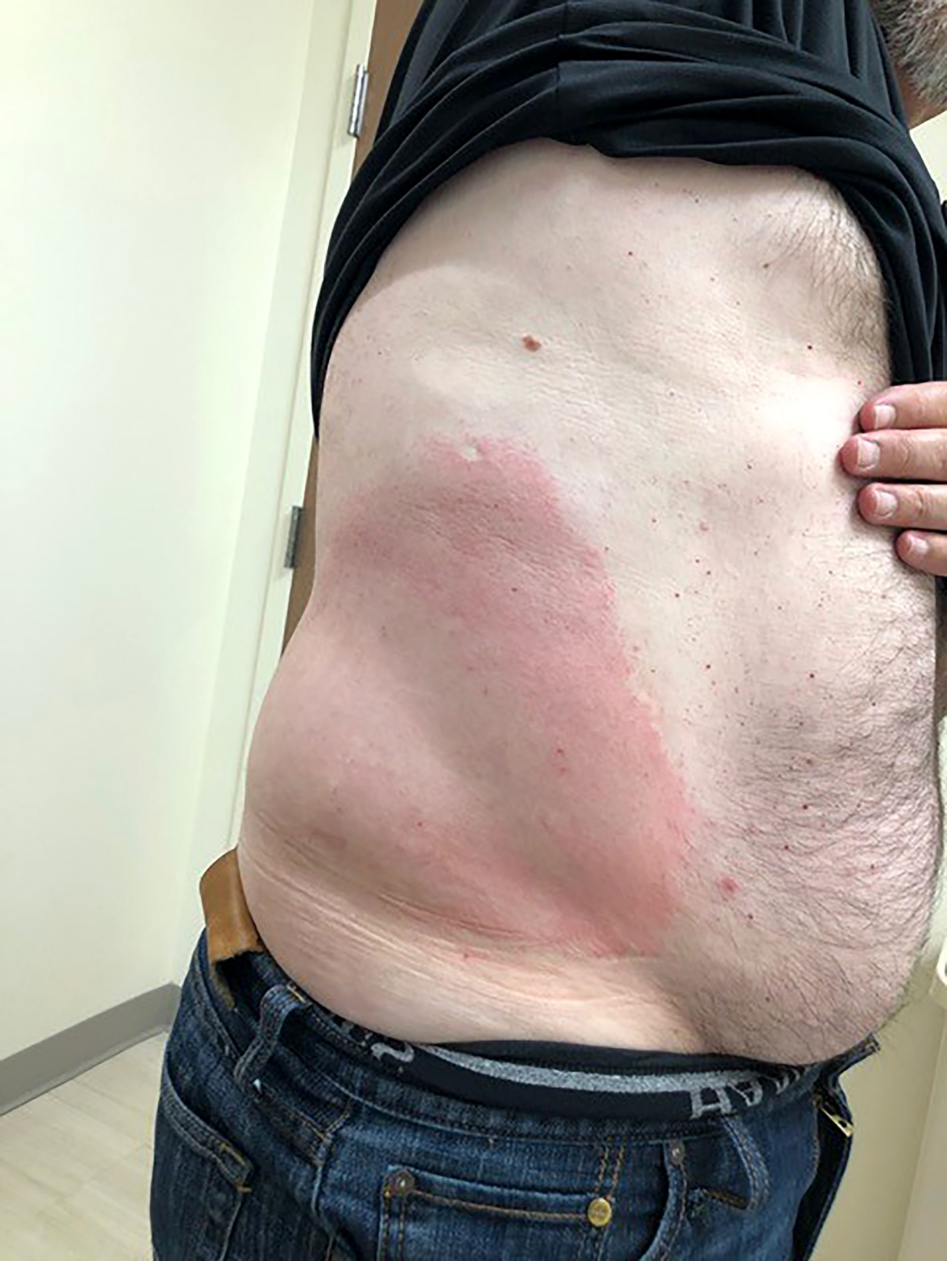
Skeeter Syndrome. The right flank of a middle-aged, male patient exhibiting Skeeter syndrome following a mosquito bite. This local area of redness and warmth was accompanied by fever. With previous episodes, he was given oral antibiotics from his primary care providers due to suspected cellulitis. This current episode responded well to topical corticosteroids and antihistamines.

Children are at increased risk of developing mosquito allergy presenting as urticaria (irregular groups of pruritic papules) and Skeeter syndrome, a type of large local inflammatory reaction (43). Skeeter syndrome involves localized redness, warmth, swelling, and pruritus following mosquito bites that can be accompanied by fever and occasionally lymphadenopathy (**Figure 2**) (44). Skeeter syndrome mimics cellulitis, but the difference is in the duration of symptoms: Skeeter syndrome occurs within hours of a mosquito bite and cellulitis has a more protracted time course. Skeeter syndrome resolves in 3-10 days and is mediated by IgE and IgG primed against mosquito saliva; it also tends to occur in immunocompromised individuals and immigrants bitten by indigenous mosquitoes without previous exposure.

Atopic children are particularly susceptible to amplified reactions. In a case-control study of 180 children, large local or unusual reactions to mosquito bites were associated with atopy (35% of cases versus 12% of controls, P < 0.001) (45). In the children with bite reactions, 32% had an accompanying atopic disease such as asthma, allergic rhinitis, or atopic dermatitis.

### Allergens involved in cross-reactivity with other arthropods

IgE-mediated allergic reactions to mosquito saliva components range from immediate or delayed large local reactions (wheals and flares) to very rare life-threatening anaphylaxis (presyncope, hypotension, and syncope) (46, 47). Less than thirty anaphylactic reactions to mosquitos have been reported worldwide, but they dramatically affect quality of life in those individuals affected (48). Of note, cross-reactivity of mosquito with other arthropods has been demonstrated; in particular, individuals with hypersensitivity to wasp venom, bees, dust mites, cockroaches, and shrimp may be susceptible to similar exaggerated reactions after *Aedes aegypti* exposure (49–51). This is due to salivary and non-salivary homologues including tropomyosin, odorant binding protein, mitochondrial cytochrome C, peptidyl-prolyl cis-trans isomerase, and protein with hypothetical magnesium ion binding function (48, 51). For patients exhibiting mosquito allergy, allergologic workup can include IgE against these potential cross-reactive allergens, although no evidence currently exists to support this testing.

### Epstein-barr virus

A triad of hypersensitivity to mosquito bites (HMB), infection with EBV, and natural killer (NK) cell proliferative disorder [coined HMB-EBV-NK (HEN) disease when coexisting in a single patient] presents with an exaggerated, local response to mosquito bites manifesting as bulla, ulceration, or necrosis. This response is initiated by CD4 T cells and amplified reaction of NK cells to mosquito saliva (52). Infection with EBV has also been implicated in the development of novel EBV-infected NK-cell line and T cell lymphoma following mosquito bite (52–54) and in hemophagocytic lymphoma when coexisting with HMB (55).

### Wells disease

Wells Disease is an eosinophilic-driven cellulitis that causes red, violaceous, blistering lesions that are pruritic. Although the etiology of Wells Disease is unknown, previous studies have suggested that mosquito bites may cause or propagate the course of the disease as patients develop extreme reactions to mosquito bites. As CD4 T cells play a large role in response to mosquito saliva antigen exposure, CD4 T cells may contribute to the proliferation of eosinophils in the case of Wells Disease (52, 56).

### Hematologic cancer

Patients diagnosed with hematologic cancers such as chronic lymphocytic leukemia and mantle cell lymphoma have also demonstrated an exaggerated response to mosquito bites (52, 57). The response is typically characterized by pruritic erythematous papules and plaques. The leading hypothesis to this immune response similarly credits CD4 T cell proliferation in response to a mosquito bite and subsequent IL-4 production (52). There are also some reports of the development of primary cutaneous diffuse large B cell lymphomas that may be associated with mosquito bites (58).

### Human immunodeficiency virus

Patients with HIV may also be more susceptible to intense mosquito bite reactions. One skin disorder that HIV patients may experience is pruritic papular eruption, although the underlying etiology is unknown. Studies examining the levels of IgE, CD4 cell counts, and eosinophilia following insect antigen exposure showed HIV patients exhibiting a positive skin response and hypersensitivity (59, 60). Therefore, it has been proposed that pruritic papular eruption in HIV patients may be in part due to mosquito bites.

## Mosquito bite prevention and recent developments

The first-line control of mosquito bites is preventing mosquitos from successfully biting their hosts. One effective method involves reducing the mosquito population, such as by limiting the amount of standing water accessible for them to complete their life cycle. In addition, using insecticide, mosquito traps, and introducing fish into ponds to consume mosquito larvae are helpful methods (61).

Other keys to prevention include utilizing physical barriers to prevent insect access to the skin, such as by staying indoors (particularly when mosquitos are most active), using mosquito netting, and wearing protective clothing. Insect repellents can be chemical or organic agents applied to clothing or to skin (62, 63). N,N-diethyl-3-methyl-benzamide (DEET) is effective at reducing the number of insect bites when applied to skin; though generally safe, exposure to concentrated formulations, excessive inhalation, or ingestion can cause neurotoxicity and systemic toxicity, urticaria syndrome, dermatitis, and bullous eruptions (**Table 1**) (67–69).

**Table 1:** Mosquito bite repellants and their safety profile.

Repellant	Average duration	Reported adverse reactions and toxicity	Special considerations
**DEET**	5 hours	Rare: CNS involvement (lethargy, headache, seizures, disorientation, ataxia, tremors, acute encephalopathy with psychosis), allergic or cutaneous manifestations, cardiovascular effects (orthostatic hypotension, bradycardia).^a^ (62, 64)	Not recommended for children under 2 years of age. Maximum concentration of 33% in children (62)Contraindicated in individuals with urea cycle disorders (62)Pregnancy Category N^b^. Increased risk of toxicity when used in conjunction with retinoids or sunscreen
**Picaridin**	8-10 hours	Rare: skin irritation (62)	Not recommended in children under 2 years of age (62)Pregnancy Category N^b^. Odorless
**Permethrin**	6 weeks or 6 washings	Rare: conjunctivitis, numbness/tingling sensation, dermatitis, air conduction passageway irritation, headache, dizziness, fatigue, excessive salivation, muscle weakness, nausea, vomiting, and neurotoxicity (ataxia, hyperactivity, hyperthermia, seizures, paralysis). May affect male fertility or cause hepatoxicity (62, 65, 66).	Pregnancy Category B^c^
**EBAAP**	2-3 hours	Eye irritation	Odorless
**Thiamine hydrochloride**	Under investigation	None reported for topical application (further research needed)	
**Oil of lemon eucalyptus**	6 hours	Rare: skin irritation in atopic individuals (62)	Not recommended for children under 3 years of age (62)Pregnancy Category N^b^
**Citronella**	2 hours	Rare: eye irritation, skin irritation, and allergic symptoms (62)	Pregnancy Category N^b^

CNS, central nervous system; DEET, N,N-diethyl-3-methyl-benzamide (formerly N,N-diethyl-meta-toluamide); EBAAP, ethyl butylacetylaminopropionate.

a Between 1956 and 2008, there were 43 confirmed case reports of DEET toxicity: 25 with CNS involvement, 17 with allergic or dermatologic manifestations, and one with cardiovascular effects. Cutaneous manifestations include urticarial reactions and hemorrhagic vesicobullous erosions after topical exposure of 50% and stronger concentrations (50).

b This drug’s pregnancy category has not yet been classified by the FDA.

c No adverse effects demonstrated in animals.

Permethrin is a broadly used, long-lasting pyrethroid insecticide coated on fabrics and mosquito nets, particularly in malaria-endemic countries enforcing control programs to curtail disease transmission. Permethrin also has some toxicity concerns, including a reduction in male fertility parameters and reduced testosterone demonstrated in rodent studies (65) as well as hepatotoxicity (66). The widespread use of pyrethroid insecticides has gained exponential traction over the past few years as mosquitos have developed pyrethroid resistance. In a 2019 study of *Anopheles gambiae* in eight farming communities in Nigeria, resistance to permethrin occurred in up to 46% of mosquitos (70). Resistance mechanisms identified in *Aedes aegypti* includes mutations in the voltage sensitive sodium channel gene (Vssc gene) and metabolic-mediated insecticide resistance (71). Due to the developing nature of resistance, more effective vector control strategies are warranted. One method involves combining a mixture of two insecticides or an insecticide and a synergist to create novel long lasting insecticide nets (LLIN; NCT03554616).

A few mosquito repellants, including several botanicals and essential oils, are gaining exposure due to decreased toxicity, increased pleasantness of the repellant smell, and improved environmental safety. Picaridin is an effective, less toxic alternative to chemical repellants (72) and thiamine hydrochloride (TH; vitamin B1) is a newer repellant currently being investigated. A 2020 pilot study of TH demonstrating an effective dose 50 of 4.57 mg (73) was followed by an *in-vivo*, ex-vivo study in 2021 which showed that TH-loaded hydrogel is comparable to DEET in terms of action duration (74).

Graphene, consisting of a single layer of carbon atoms, has recently been studied as a lightweight, wearable technology used for chemical, mechanical, and radiative protection (75). A new study examined whether graphene-based materials may also provide protection against mosquitos (75). In the dry state, graphene was highly effective at suppressing mosquito biting behavior by interfering with host chemosensing (trapping skin-associated molecular attracts beneath) and by mechanical bite prevention.

## Therapies

When mosquito bites are inevitable, prophylaxis with second generation antihistamines can be given to reduce local skin reactions (**Table 2**). One study has suggested that this is especially true for individuals with hypersensitivity (wheals > 5 mm) (76). Oral daily dosing regimens with levocetirizine 5 mg, cetirizine 10 mg, and rupatadine 10 mg have been proven through placebo-controlled trials to decrease both size of whealing and skin pruritus in adults (77–79). Loratadine (0.3 mg/kg) in children likewise significantly decreased wheal size by 45% (P < 0.001, 25 children) and pruritus by 78% (P = 0.011, 12 children) (80). These medications can relieve both immediate and delayed allergic symptoms measured 12 and/or 24 hours afterwards. In a trial comparing cetirizine, ebastine, and loratadine 10 mg, Karppinen et al. showed cetirizine and ebastine were effective in decreasing wheal size by 30-40% and pruritus by 70–80% compared to placebo, with cetirizine improving itch to the greatest extent; however, cetirizine also induced the highest frequency of sedation (81). Of note, ebastine and rupatadine are not approved for use in the United States.

**Table 2:** Second-line mosquito bite therapies.

Topical Treatments
Calamine lotion Menthol-Camphor Local anesthetic (pramoxine, lidocaine, benzocaine, lidocaine/prilocaine) Antihistamines Corticosteroids Cold compresses Homeopathic after-bite gel Other home remedies, such as sodium bicarbonate
Oral Treatments
Antihistamines^a^ Glucocorticoids Leukotriene receptor antagonists
Other Treatments
Intralesional corticosteroids Epinephrine (anaphylaxis) Immunotherapy Omalizumab (off-label) Suction tools Electronic heat device

a Supported efficacy for mosquito bite reactions through double-blind, placebo-controlled trials.

When mosquito bites do occur, treatment is aimed at alleviating pruritus through topical applications of medications and medication alternatives as well as oral antihistamines as described above. Topical therapies’ target of action involves inhibiting the pruritic pathways or providing local anesthetic. For example, clinical studies have demonstrated decreased itch after repeated noxious heat, which is thought to activate transient receptor potential cation channel subfamily V member 1 (TRPV1), influencing proteinase-activated receptor-2 (PAR-2) carrying C-fibers and decreasing pruritus (82–84). A medical device utilizing local electrical discharges to generate skin warmth showed a statistically significant improvement in pruritus compared to placebo (19 of 27 patients reported at least a 40% improvement in itch) lasting up to 24 hours (85). Conversely, cooling is also shown to reduce itch through TRPM8, an ion channel expressed in peripheral afferent nerve endings (86). When menthol or cold activates the TRPM8 receptor, inhibition of both histaminergic and non-histaminergic itch pathways occurs.

Other options with a paucity of supporting evidence for specific alleviation of mosquito bite symptoms include topical therapy with glucocorticoids, calamine lotion, pramoxine or lidocaine, and other homeopathic remedies such as plant extracts (Echinacea angustifolia mother tincture, Ledum palustre D1 mother tincture, and Urtica Urens mother tincture) (87) and sodium bicarbonate. Interestingly, topical glucocorticoids, which broadly target the inflammatory pathways of itch and in the authors’ opinion are effective, have not been subjected to controlled clinical trials for mosquito-related itch. Oral leukotriene receptor antagonists should also be explored further in clinical trials (1). Suction tools are designed to remove mosquito saliva, though no randomized, blinded clinical trials have evaluated their effectiveness. In the rare instances of severe local or systemic mosquito bite reactions, oral glucocorticoids may be indicated, as they are for other urticarias (1).

Finally, several trials studying immunotherapy with whole-body mosquito extract have revealed promising results, but insufficient evidence. A double blind, randomized, placebo-controlled trial demonstrated significant improvement in 40 patients receiving immunotherapy for one year compared to baseline and to the placebo group (88). Improvement was observed for skin reactions, symptom scores for rhinitis and asthma, and forced expiratory volume in one second, showing that mosquito immunotherapy is beneficial for both allergic rhinitis and bronchial asthma. Within the treatment group, serologic analysis showed a nonsignificant decrease in IgE (P = 0.02), significant elevation in IgG4 levels (P = .001), and a significant decrease in the IgE/IgG4 ratio (P = .001). In another study of two patients exhibiting anaphylactic episodes, immunotherapy resolved symptoms in one and decreased reactions in the other (89). Desensitization with immunotherapy may be considered for future patients with anaphylactic reactions, but it is not currently available and further studies with randomization, a control group, and blinding are needed to fully assess the effects of this intervention (12, 90–93). Additionally, future studies are needed utilizing salivary extracts in leu of whole-body mosquito extracts, as several mosquito saliva proteins have been identified as allergenic (31).

An available off-label alternative to anaphylaxis prevention may be found in omalizumab, an anti‐IgE monoclonal antibody. One case report of a patient with systemic anaphylaxis to mosquito manifesting as urticaria, presyncope, and hypotension reported neither anaphylaxis nor mild local reactions following mosquito bites after receiving 300 mg of omalizumab subcutaneously every 4 weeks for 3 months (48).

Interestingly, several studies have demonstrated that the local microenvironment induced by mosquito saliva facilitates the transmission of mosquito-borne diseases, providing an opportunity for therapeutic intervention. A few mechanisms have been identified. Mosquito saliva components reduce the host’s antiviral Th1 immune response, permitting easier entry and replication of viruses in the host (94–96). Anti-saliva peptide antibodies provoked through vaccination could enable a more robust Th1 response and interferon release with dampened leukocyte influx to the inoculation site and subsequent decrease in cell trafficking to draining lymph nodes, thus lessening the ease with which the pathogen spreads systemically (97). Vaccines against mosquito saliva peptides are undergoing clinical assessment to determine whether they will be safe and useful for the prevention of pathogen transmission (98).

## Conclusions

Mosquito bite-induced pruritus is caused by local reactions to mosquito saliva components. While the exact pathophysiology isn’t well-understood, the reaction is thought to be mediated in the majority of cases by histamine release either from the saliva and/or IgE-mediated hypersensitivity reactions. IgE-independent pathways are associated with delayed reactions. However, other non-histaminergic itch mediators such as leukotrienes, proteases, and type 2 cytokines may have a role. Because mosquitos are ubiquitous with a significant induction of worldwide disease burden, it is critical for healthcare providers to recognize how to prevent and treat these mosquito bites. Treatment consists of second-generation antihistamines and topical steroids, and further research on topical pharmaceuticals is needed. Failure to treat the pruritus can result in secondary pigment changes, scarring, and infections.

## Author contributions

AV and AL drafted the article. GY critically revised the article. All authors contributed to the article and approved the submitted version.

## Conflict of interest

GY conducted clinical trials or received honoraria for serving as a member of the Scientific Advisory Board and consultant of Pfizer, TREVI, Regeneron, Sanofi, Galderma, Novartis, Bellus, Kiniksa, and Eli Lilly and received research funds from Pfizer, Leo, Sanofi, Regeneron, Eli Lilly, and Novartis.

The remaining authors declare that the research was conducted in the absence of any commercial or financial relationships that could be construed as a potential conflict of interest.

## Publisher’s note

All claims expressed in this article are solely those of the authors and do not necessarily represent those of their affiliated organizations, or those of the publisher, the editors and the reviewers. Any product that may be evaluated in this article, or claim that may be made by its manufacturer, is not guaranteed or endorsed by the publisher.

## Abbreviations

CNS, central nervous system; DEET, N,N-diethyl-3-methyl-benzamide (formerly N,N-diethyl-meta-toluamide); EBAAP, ethyl butylacetylaminopropionate; EBV, Epstein-Barr virus; HIV, human immunodeficiency virus; HLA, human leukocyte antigen; IL, interleukin; HEN, hypersensitivity to mosquito bites-Epstein-Barr virus-natural killer; HMB, hypersensitivity to mosquito bites; NK, natural killer; PAR-2, proteinase-activated receptor-2; TH, thiamine hydrochloride; TRP, transient receptor potential.
